# Short-term outcomes of extremely preterm infants at discharge: a multicenter study from Guangdong province during 2008–2017

**DOI:** 10.1186/s12887-019-1736-8

**Published:** 2019-11-04

**Authors:** Fan Wu, Guosheng Liu, Zhoushan Feng, Xiaohua Tan, Chuanzhong Yang, Xiaotong Ye, Yiheng Dai, Weiyi Liang, Xiuzhen Ye, Jing Mo, Lu Ding, Benqing Wu, Hongxiang Chen, Chiwang Li, Zhe Zhang, Xiao Rong, Wei Shen, Weimin Huang, Bingyan Yang, Junfeng Lv, Leying Huo, Huiwen Huang, Hongping Rao, Wenkang Yan, Yong Yang, Xuejun Ren, Fangfang Wang, Dong Liu, Shiguang Diao, Xiaoyan Liu, Qiong Meng, Yu Wang, Bin Wang, Lijuan Zhang, Yuge Huang, Dang Ao, Weizhong Li, Jieling Chen, Yanling Chen, Wei Li, Zhifeng Chen, Yueqin Ding, Xiaoyu Li, Yuefang Huang, Niyang Lin, Yangfan Cai, Shasha Han, Ya Jin, Zhonghe Wan, Yi Ban, Bo Bai, Guanghong Li, Yuexiu Yan, Qiliang Cui

**Affiliations:** 1 Department of Neonatology, the First Affiliated Hospital of Jinan University, Guangzhou, 510630 Guangdong China; 2 Department of Pediatrics, the Third Affiliated Hospital of Guangzhou Medical University, Guangzhou, 510150 Guangdong China; 3 Department of Neonatology, Shenzhen Maternal & Child Healthcare Hospital, Affiliated Southern Medical University, Shenzhen, 518028 Guangdong China; 4 Department of Neonatology, Foshan Maternal and Child’s Hospital, Foshan, 528000 Guangdong China; 5 Department of Neonatology, Women and Children Hospital of Guangdong Province, Guangzhou, 510010 Guangdong China; 6 Department of Neonatology, Shenzhen People’s Hospital, Shenzhen, 518020 Guangdong China; 7 Department of Neonatology, Meizhou People’s Hospital, Meizhou, 514031 Guangdong China; 8 Department of Neonatology, Guangzhou Women and Children’s Medical Center, Guangzhou, 510120 Guangdong China; 9 Department of Neonatology, Nanfang Hospital of Southern Medical University, Guangzhou, 510515 Guangdong China; 10 Department of Neonatology, Boai Hospital of Zhongshan, Zhongshan, 528400 Guangdong China; 11 Department of Neonatology, Zhuhai Maternity and Child Health Hospital, Zhuhai, 519001 Guangdong China; 12 Department of Neonatology, Huizhou Municipal Central Hospital, Huizhou, 516001 Guangdong China; 13 Department of Neonatology, Dongguan Maternity and Child Health Hospital, Dongguan, 523002 Guangdong China; 14 Department of Neonatology, Jiangmen Central Hospital, Affiliated Jiangmen Hospital of Sun Yat-sen University, Jiangmen, 529000 Guangdong China; 15 Department of Neonatology, Yuebei People’s Hospital, Shaoguan, 512026 Guangdong China; 16 Department of Neonatology, Guangdong Second Provincial People’s Hospital, Guangzhou, 510317 Guangdong China; 17 Department of Pediatrics, Zhujiang Hospital of Southern Medical University, Guangzhou, 510280 Guangdong China; 18 Department of Pediatrics, the Affiliated Hospital of Guangdong Medical University, Zhanjiang, 524001 Guangdong China; 19 Department of Neonatology, the Second Affiliated Hospital of Shantou University Medical College, Shantou, 515041 Guangdong China; 20 Department of Neonatology, Jinan University Medical College Affiliated Dongguan Hospital, Dongguan, 523900 Guangdong China; 21 Department of Pediatrics, Dongguan People’s Hospital, Dongguan, 523000 Guangdong China; 22 Department of Neonatology, the First Affiliated Hospital of Sun Yat-sen University, Guangzhou, 510080 Guangdong China; 23 Department of Neonatology, the First Affiliated Hospital of Shantou University Medical College, Shantou, 515041 Guangdong China; 24 Department of Neonatology, Nanhai District People’s Hospital of Foshan, Foshan, 528200 Guangdong China; 25 Department of Neonatology, Huadu District People’s Hospital of Guangzhou, Guangzhou, 510800 Guangdong China; 26 Department of Pediatrics, the First People’s Hospital of Zhaoqing, Zhaoqing, 526020 Guangdong China

**Keywords:** Extremely preterm infant, Low birth weight, Survival rate, Complication, Outcome, China

## Abstract

**Background:**

An increasing number of extremely preterm (EP) infants have survived worldwide. However, few data have been reported from China. This study was designed to investigate the short-term outcomes of EP infants at discharge in Guangdong province.

**Methods:**

A total of 2051 EP infants discharged from 26 neonatal intensive care units during 2008–2017 were enrolled. The data from 2008 to 2012 were collected retrospectively, and from 2013 to 2017 were collected prospectively. Their hospitalization records were reviewed.

**Results:**

During 2008–2017, the mean gestational age (GA) was 26.68 ± 1.00 weeks and the mean birth weight (BW) was 935 ± 179 g. The overall survival rate at discharge was 52.5%. There were 321 infants (15.7%) died despite active treatment, and 654 infants (31.9%) died after medical care withdrawal. The survival rates increased with advancing GA and BW (*p* < 0.001). The annual survival rate improved from 36.2% in 2008 to 59.3% in 2017 (*p* < 0.001). EP infants discharged from hospitals in Guangzhou and Shenzhen cities had a higher survival rate than in others (*p* < 0.001). The survival rate of EP infants discharged from general hospitals was lower than in specialist hospitals (*p* < 0.001). The major complications were neonatal respiratory distress syndrome, 88.0% (1804 of 2051), bronchopulmonary dysplasia, 32.3% (374 of 1158), retinopathy of prematurity (any grade), 45.1% (504 of 1117), necrotizing enterocolitis (any stage), 10.1% (160 of 1588), intraventricular hemorrhages (any grade), 37.4% (535 of 1431), and blood culture-positive nosocomial sepsis, 15.7% (250 of 1588). The multivariate logistic regression analysis indicated that improved survival of EP infants was associated with discharged from specialist hospitals, hospitals located in high-level economic development region, increasing gestational age, increasing birth weight, antenatal steroids use and a history of premature rupture of membranes. However, twins or multiple births, Apgar ≤7 at 5 min, cervical incompetence, and decision to withdraw care were associated with decreased survival.

**Conclusions:**

Our study revealed the short-term outcomes of EP infants at discharge in China. The overall survival rate was lower than the developed countries, and medical care withdrawal was a serious problem. Nonetheless, improvements in care and outcomes have been made annually.

## Background

Extremely preterm (EP) infants with gestational age (GA) <28 weeks are at high risk of morbidity and mortality. Due to prematurity, they suffer higher incidence rate in respiratory distress syndrome (RDS), necrotizing enterocolitis (NEC), intraventricular hemorrhage (IVH), bronchopulmonary dysplasia (BPD), retinopathy of prematurity (ROP), infection and so on. Most of them need to receive advanced life support during the first several days. Consequently, the long-term survival rates were usually below 10% before 1970 [1]. And, approximately one-quarter of EP infants born in the 1990s had a major disability at preschool age, such as impaired mental development, cerebral palsy, blindness, or deafness [2]. In recent decades, the outcomes of EP infants have been greatly improved due to tremendous advancement in perinatal care including antenatal steroids, surfactant replacement therapy, mechanical ventilation, nutrition therapy and increase of active treatment. From 2003 to 2007, the median survival rates for infants born at 22–27 weeks of gestation were 6, 26, 55, 72, 84 and 88%, respectively,in the United States [3]. Other studies from Netherlands, England, Canada, Switzerland and Australia demonstrated similar improvements as GA increased [4–8]. Obviously, these studies were completed in developed countries. However, outcomes of EP infants in China, a developing country with the greatest population, still remain unclear.

The available epidemiological data including short-term and long-term outcomes of EP infants are very important to family counseling, clinical practice and social policies. Nevertheless, a population-based survey of EP infants in China is quite difficult to conduct because the exact number of EP infants is hard to evaluate. An infant born below 28 weeks of gestation is defined as an abortus, not as an infant, according to the current definition of preterm birth in China [9, 10]. The number of EP infants died immediately in the delivery room due to lack of active and effective resuscitation, cannot be counted. Only those transferred to neonatal intensive care units (NICUs) after resuscitation can be known exactly. Thus, in this study, we focused solely on the short-term outcomes at discharge of EP infants from 26 NICUs in Guangdong province.

## Methods

### Participating centers

The collaborative study group composed of 26 NICUs was established before data collection. These NICUs were located in four regions of Guangdong province which were representative of medical units offering neonatal intensive care in the irrespective areas. The Third Affiliated Hospital of Guangzhou Medical University was responsible for coordinating this survey, where all the data were aggregated, stored and analyzed. This study was approved by the Ethics Committees of the Third Affiliated Hospital of Guangzhou Medical University. The same diagnostic criteria were applied to all NICUs.

### Subjects and data collection

All EP infants discharged from our collaborative NICUs were studied, whereas live-born infants not admitted to NICUs and still births in the delivery room were not enrolled. The study protocol was fully discussed by all members, and a standardized questionnaire for data collection including maternal and neonatal demographics, major complications, treatments and outcomes was designed. In fact, this study was initiated at the end of 2012 and is still ongoing at present. Therefore, the data from January 1, 2008 to December 31, 2012 were collected retrospectively, and data from January 1, 2013 to December 31, 2017 were collected prospectively. The relevant records of all enrolled infants and their mothers were reviewed and filled in the questionnaire. All sheets were sent to the Third Affiliated Hospital of Guangzhou Medical University and the data from each questionnaire were input into the database. In order to minimize bias among centers and investigators, comprehensive and systematic training was provided to the staffs involved in the survey. Data collected by the researcher at each collaborative NICU was supervised and checked by the NICU director, who was responsible for the quality assurance. Meanwhile, these records were also checked for accuracy and completeness by collaborative centers.

### Definitions and classifications

In this study, survivors were defined as neonates who survived to the time of discharge. GA was calculated from the date of the last menstrual period or was determined by fetal ultrasound assessment. RDS was diagnosed in preterm infants with the onset of respiratory distress shortly after birth and a compatible chest radiograph appearance [11]. BPD was defined as oxygen dependency at 36 weeks of corrected age or at discharge [12]. The criteria utilized in our survey for the diagnosis of NEC and for grading the severity of disease were based on Bell’s stage [13]. ROP and the graded standard were defined by the international classification of ROP [14]. IVH and periventricular leukomalacia (PVL) were diagnosed by cranial ultrasonography or magnetic resonance imaging (MRI). The Papile grading system was used to grade IVH [15], and PVL was defined as degeneration of white matter adjacent to the cerebral ventricles following cerebral hypoxia or brain ischemia [16]. Nosocomial sepsis was defined as blood culture-positive sepsis occurring beyond 48 h of life [17].

### Statistical analysis

All statistical analyses were performed using SPSS 18.0 for Windows (IBM, Armonk, NY, USA). Continuous variables were shown as means ± standard deviation (SD) or as medians (P25, P75) when their distributions were highly skewed, which were analyzed using t-tests or Mann-Whitney tests. Categorical variables arepresented as rates and odds ratio with 95% confidence intervals (CI), which were analyzed using Chi-square tests. Multivariate analyses were performed by using logistic regression to analyze the risk factors of survival in preterm infants. *P* < 0.05 was considered statistically significant.

## Results

### Demographics of EP infants and mothers

During the 10-year survey, in total, 2051 EP infants were enrolled. The overall survival rate at discharge was 52.5% (1076 of 2051). The smallest GA and lowest BW in survivors were 22 weeks and 480 g respectively. Totally, the mean GA was 26.68 ± 1.00 weeks, and the distribution ranged from 26 (1.3%) for less than 24 weeks, 105 (5.1%) for 24 weeks, 263 (12.8%) for 25 weeks, 548 (26.7%) for 26 weeks to 1109 (54.1%) for 27 weeks. The mean BW was 935 ± 179 g, and the distribution ranged from 16 (0.8%) for less than 500 g, 36 (1.8%) for 500 - 599 g, 106 (5.2%) for 600 - 699 g, 255 (12.4%) for 700 - 799 g, 398 (19.4%) for 800 - 899 g, 495 (24.1%) for 900 - 999 g to 745 (36.3%) for equal or more than 1000 g. In order to clarify the current treatment and outcome of EP infants, we specifically grouped the EP infants based on survival or not, as it was presented in Table 1. The overall mean GA or BW in the survivor group was greater than in the non-survivor group (*p* < 0.001). There was no significant difference in the sex ratio between the survivor and non-survivor group. There were fewer infants with Apgar scores ≤ 3 at 1 min and ≤ 7 at 5 min in the survivor group (both *p* < 0.001). Comparing with the non-survivor group, the survivor group had higher rates of receiving surfactant therapy (*p* < 0.001) and non-invasive ventilation (*p* < 0.001), but the rate of requiring mechanical ventilation was lower (*p* < 0.01). Even more, there were significant differences in the days of mechanical ventilation, non-invasive ventilation, oxygen therapy and hospital stay between the two groups (*p* < 0.001).

**Table 1:** Demographics of extremely preterm (EP) infants and the mothers in outcome categories

Characteristics	Survivors (*N* = 1076)	Non-survivors (*N* = 975)	Total (*N* = 2051)	*p*-value	*OR (95% CI)*
Characteristics of infants					
GA (weeks), mean ± SD	26.92 ± 0.86	26.41 ± 1.07	26.68 ± 1.00	<0.001	/
BW (grams), mean ± SD	976 ± 167	889 ± 180	935 ± 179	<0.001	/
Gender (male), n (%)	669 (62.2)	586 (60.1)	1255 (61.2)	>0.05	1.091 (0.913–1.303)
Apgar score, n (%)					
≤ 3 at 1 min	95 (8.8)	168 (17.2)	263 (12.8)	<0.001	0.465 (0.356–0.608)
4**~**7 at 1 min	369 (34.3)	391 (40.1)	760 (37.1)	<0.01	0.780 (0.651–0.933)
≤ 3 at 5 min	9 (0.8)	43 (4.4)	52 (2.5)	<0.001	0.183 (0.089–0.377)
4**~**7 at 5 min	130 (12.1)	214 (21.9)	344 (16.8)	<0.001	0.489 (0.385–0.620)
Surfactant therapy, n (%)	900 (83.6)	718 (73.6)	1618 (78.9)	<0.001	1.830 (1.476–2.270)
Surfactant therapy twice or more, n (%)	155 (14.4)	155 (15.9)	310 (15.1)	>0.05	0.890 (0.699–1.134)
Mechanical ventilation, n (%)	816 (75.8)	787 (80.7)	1603 (78.2)	<0.01	0.750 (0.607–0.926)
Days of mechanical ventilation (days), median (P25, P75)	8.0 (2.0,22.0)	2.3 (1.0,7.3)	4.2 (1.0,15.0)	<0.001	/
Non-invasive ventilation, n (%)	880 (81.8)	240 (24.6)	1120 (54.6)	<0.001	13.750 (11.117–17.007)
Days of non-invasive ventilation (days), median (P25, P75)	15.4 (6.0,26.0)	3.1 (1.0,8.0)	12.0 (4.0,23.0)	<0.001	/
Total days of oxygen therapy (days), median (P25, P75)	42.7 (25.0,60.6)	3.0 (1.0,9.8)	19.5 (2.8,47.0)	<0.001	/
Length of hospital stay (days), median (P25, P75)	71.0 (55.5,87.0)	3.0 (1.0,10.0)	35.0 (3.0,73.0)	<0.001	/
Characteristics of mothers					
History of pregnancy problems^a^, n (%)	412 (38.3)	349 (35.8)	761 (37.1)	>0.05	1.113 (0.930–1.332)
Age ≥ 35 years, n (%)	238 (22.1)	177 (18.2)	415 (20.2)	<0.05	1.280 (1.030–1.591)
Cesarean section, n (%)	221 (20.5)	172 (17.6)	393 (19.2)	>0.05	1.207 (0.967–1.506)
Twin/multiple pregnancy, n (%)	392 (36.4)	404 (41.4)	796 (38.8)	<0.05	0.810 (0.678–0.968)
Antenatal steroids, n (%)	609 (56.6)	367 (37.6)	976 (47.6)	<0.001	2.160 (1.810–2.579)
Dexamethasone ≥ 4 doses	390 (36.2)	204 (20.9)	594 (29.0)	<0.001	2.149 (1.762–2.620)
Dexamethasone 1~3 dose(s)	219 (20.4)	163 (16.7)	382 (18.6)	<0.05	1.273 (1.017–1.593)
Premature rupture of membranes, n (%)	305 (28.3)	194 (19.9)	499 (24.3)	<0.001	1.593 (1.297–1.956)
Infection in the middle trimester of pregnancy, n (%)	82 (7.6)	66 (6.8)	148 (7.2)	>0.05	1.136 (0.812–1.590)
Gestational diabetes mellitus, n (%)	99 (9.2)	88 (9.0)	187 (9.1)	>0.05	1.021 (0.756–1.380)
Pregnancy induced hypertension syndrome, n (%)	71 (6.6)	80 (8.2)	151 (7.4)	>0.05	0.790 (0.567–1.102)
Placental abruption/Placenta previa, n (%)	72 (6.7)	81 (8.3)	153 (7.5)	>0.05	0.792 (0.569–1.101)
Thyroid dysfunction, n (%)	31 (2.9)	22 (2.3)	53 (2.6)	>0.05	1.285 (0.739–2.235)
Cervical incompetence, n (%)	28 (2.6)	48 (4.9)	76 (3.7)	<0.01	0.516 (0.321–0.829)

*P*-value means the contrast between survivors and non-survivors

*GA* gestational age, *BW* birth weight, *SD* standard deviation, *P25* the 25th percentile, *P75* the 75th percentile, *OR* odds ratio, *CI* confidence interval

^a^History of pregnancy problems refer to that the mother had at least one of the histories as follow: spontaneous abortion, induced abortion, stillbirth, preterm birth, ectopic pregnancy, or baby died during the neonatal period

For the mothers, there was a similar incidence in the history of pregnancy problems between the survivor and non-survivor groups. Comparing with the mothers in the non-survivor group, the mothers in the survivor group had a higher proportion of age ≥ 35 years (*p* < 0.05) and antenatal steroids therapy (*p* < 0.001), while a lower rate of twin/multiple pregnancies (*p* < 0.05). Interestingly, the mothers in the survivor group had a higher incidence of premature rupture of membranes (*p* < 0.001) and a lower incidence of cervical incompetence (*p* < 0.01). However, there was no significant difference in the rate of cesarean section, or in the incidences of infection in the middle trimester of pregnancy, gestational diabetes mellitus, pregnancy-induced hypertension syndrome, placental abruption/placenta previa or thyroid dysfunction.

### Increase in number and survival rates of EP infants from 2008 to 2017

The number of EP infants discharged from NICUs increased sharply from 58 cases in 2008 to 408 cases in 2017, as it was shown in Table 2. The survival rate improved annually from 36.2% in 2008 to 59.3% in 2017 (*p* < 0.001). The ratio of EP infants to all preterm infants rose annually from 0.70% in 2008 to 2.29% in 2017 (*p* < 0.001). Similarly, the ratio of EP infants to all discharged infants increased annually from 0.17% in 2008 to 0.67% in 2017 (*p* < 0.001).

**Table 2:** The survival rate of extremely preterm (EP) infants at discharge from 2008 to 2017

	2008	2009	2010	2011	2012	2013	2014	2015	2016	2017	Total
All EP infants, n	58	75	78	158	163	186	257	299	369	408	2051
Survived, n (%)	21 (36.2)	25 (33.3)	28 (35.9)	66 (41.8)	79 (48.5)	95 (51.1)	130 (50.6)	170 (56.9)	220 (59.6)	242 (59.3)	1076 (52.5)
Died despite active treatment, n (%)	14 (24.1)	16 (21.3)	12 (15.4)	33 (20.9)	16 (9.8)	22 (11.8)	54 (21.0)	58 (19.4)	52 (14.1)	44 (10.8)	321 (15.7)
Died after medical care withdrawal, n (%)	23 (39.7)	34 (45.3)	38 (48.7)	59 (37.3)	68 (41.7)	69 (37.1)	73 (28.4)	71 (23.7)	97 (26.3)	122 (29.9)	654 (31.9)
All preterm infants, n (%)^*^	8335 (0.70)	8499 (0.88)	10,795 (0.72)	14,210 (1.11)	15,611 (1.04)	16,574 (1.12)	16,266 (1.58)	15,967 (1.87)	18,417 (2.00)	17,780 (2.29)	142,454 (1.44)
All infants, n (%)^#^	33,299 (0.17)	33,328 (0.22)	43,305 (0.18)	53,066 (0.29)	58,718 (0.28)	55,603 (0.33)	53,951 (0.48)	53,937 (0.55)	60,075 (0.61)	60,886 (0.67)	506,168 (0.41)

R × C Chi-square test (linear-by-linear association) showed that the ratios of EP infants in all preterm infants and in all infants discharged rose up annually (both *P*<0.001). And the annual survival rate of EP infants improved from 2008 to 2017 (*P*<0.001). EP: Extremely preterm; ^*^Percentile of EP infants discharged in all preterm infants discharged; ^#^Percentile of EP infants discharged in all infants discharged

### Improved survival rates of EP infants with increasing GA and BW from 2008 to 2017

For the entire cohort, with the increase in GA or BW, the number of EP infants increased from 26 (GA < 24 weeks) or 52 (BW < 600 g) to 1109 (GA = 27 weeks) or 745 (BW ≥ 1000 g), as it was displayed in Table 3. As GA increased from less than 24 weeks to 27 weeks, the survival rate improved from 11.5 to 62.4% (*p* < 0.001). As BW increased from less than 600 g to above 1000 g, the survival rate rose dramatically from 23.1 to 64.2% (*p* < 0.001). There was only one infant who survived with BW less than 500 g.However, there were no significant differences in GA or BW between 2008–2012 and 2013–2017 (*p* = 0.073 or *p* = 0.500). The mean GA or BW for EP infants during 2008–2012 were 26.73 ± 0.93 weeks or 935 ± 172 g respectively, while during 2013–2017 were 26.66 ± 1.02 weeks or 935 ± 181 g respectively.

**Table 3:** The survival rate of extremely preterm (EP) infants in relation to gestational age and birth weight

	GA (weeks)					BW (grams)					
	<24	24~	25~	26~	27~	<600	600~	700~	800~	900~	≥1000
All EP infants, n	26	105	263	548	1109	52	106	255	398	495	745
Survived, n (%)	3 (11.5)	36 (34.3)	89 (33.8)	256 (46.7)	692 (62.4)	12 (23.1)	32 (30.2)	86 (33.7)	186 (46.7)	282 (57.0)	478 (64.2)
Died despite active treatment, n (%)	8 (30.8)	23 (21.9)	56 (21.3)	90 (16.4)	144 (13.0)	11 (21.2)	33 (31.1)	41 (16.1)	66 (16.6)	74 (14.9)	96 (12.9)
Died after medical care withdrawal, n (%)	15 (57.7)	46 (43.8)	118 (44.9)	202 (36.9)	273 (24.6)	29 (55.8)	41 (38.7)	128 (50.2)	146 (36.7)	139 (28.1)	171 (23.0)

R × 2 Chi-square test (linear-by-linear association) showed that the survival rate of EP infants improved with increasing GA and BW (both *P*<0.001). *EP* extremely preterm, *GA* gestational age, *BW* birth weight

### Variations in survival rates among different regions and hospitals

According to the level of economic development where the hospitals were located, our collaborative NICUs could be divided into three categories, specifically involving Guangzhou and Shenzhen (including 11 hospitals) with high level, the cities in the Pearl Delta (including 10 hospitals) with medium level and the cities outside the Pearl Delta (including 5 hospitals) with low level. As it was shown in Table 4, from 2008 to 2017, the overall survival rates were positively correlated with the level of regional economic development, and there were significant differences among these regions (*p* < 0.001). From the low to high level, the mean GA of EP infants was 26.74 ± 0.90 weeks, 26.79 ± 0.92 weeks and 26.62 ± 1.04 weeks, which showed a significant difference (*p* = 0.002); while the mean BW was 920 ± 174 g, 947 ± 170 g and 932 ± 183 g, which showed no significant difference (*p* = 0.104).

**Table 4:** Differences of survival rate among regions and between hospitals

	Economic development levels of the region			Types of hospital	
	High-level	Middle-level	Low-level	General hospitals	Maternal and children’s hospitals
All EP infants, n	1274	559	218	1083	968
Survived, n (%)	708 (55.6)	281 (50.3)	87 (39.9)	488 (45.1)	588 (60.7)
Died despite active treatment, n (%)	182 (14.3)	81 (14.5)	58 (26.6)	213 (19.7)	108 (11.2)
Died after medical care withdrawal, n (%)	384 (30.1)	197 (35.2)	73 (33.5)	382 (35.3)	272 (28.1)

R × 2 Chi-square test (linear-by-linear association) showed that the overall survival rate of EP infants discharged from different regions improved with the ascending level of economic development (*P*<0.001). And 2 × 2 Chi-square test showed that the survival rate of EP infants discharged from general hospitals was lower than those from maternal and children’s hospitals (*P*<0.001). EP: Extremely preterm

Among the 26 hospitals, 19 were general hospitals and the others were specialist hospitals (maternal and children’s hospitals), and the overall survival rate of EP infants discharged from general hospitals was lower than specialist hospitals (OR = 0.530, 95% CI: 0.445–0.632, *p* < 0.001). Nevertheless, the mean GA and BW of EP infants from general hospitals (26.75 ± 0.94 weeks and 944 ± 177 g respectively) were both greater than those from specialist hospitals (26.60 ± 1.05 weeks and 924 ± 180 g respectively), and there were significant differences (*p* = 0.001 and *p* = 0.013).

### Complications of EP infants

**Fig. 1:**
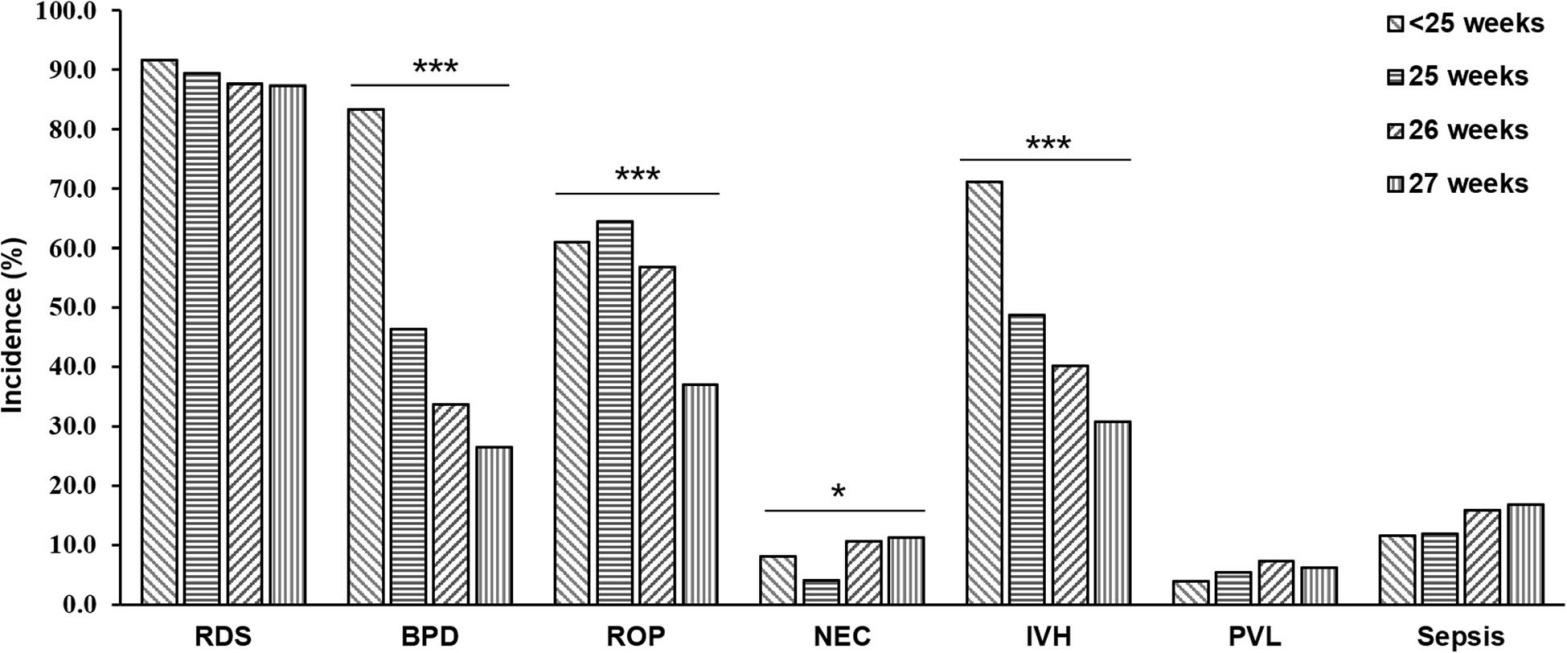
The incidence of complications at each gestational age (GA) group. R × 2 Chi-square test (linear-by-linear association) showed that the incidence of BPD, ROP, IVH decreased with increasing GA (all *p*<0.001), whereas the incidence of NEC rose up (*p*<0.05). There were no significant differences in the incidence of RDS, PVL, and Sepsis. RDS: Neonatal respiratory distress syndrome; BPD: Bronchopulmonary dysplasia; ROP: Retinopathy of prematurity; NEC: Necrotizing enterocolitis; IVH: Intraventricular hemorrhage; PVL: Periventricular leukomalacia. Sepsis: blood culture-positive nosocomial sepsis.*:*p*<0.05; ***: *p*<0.001

**Fig. 2:**
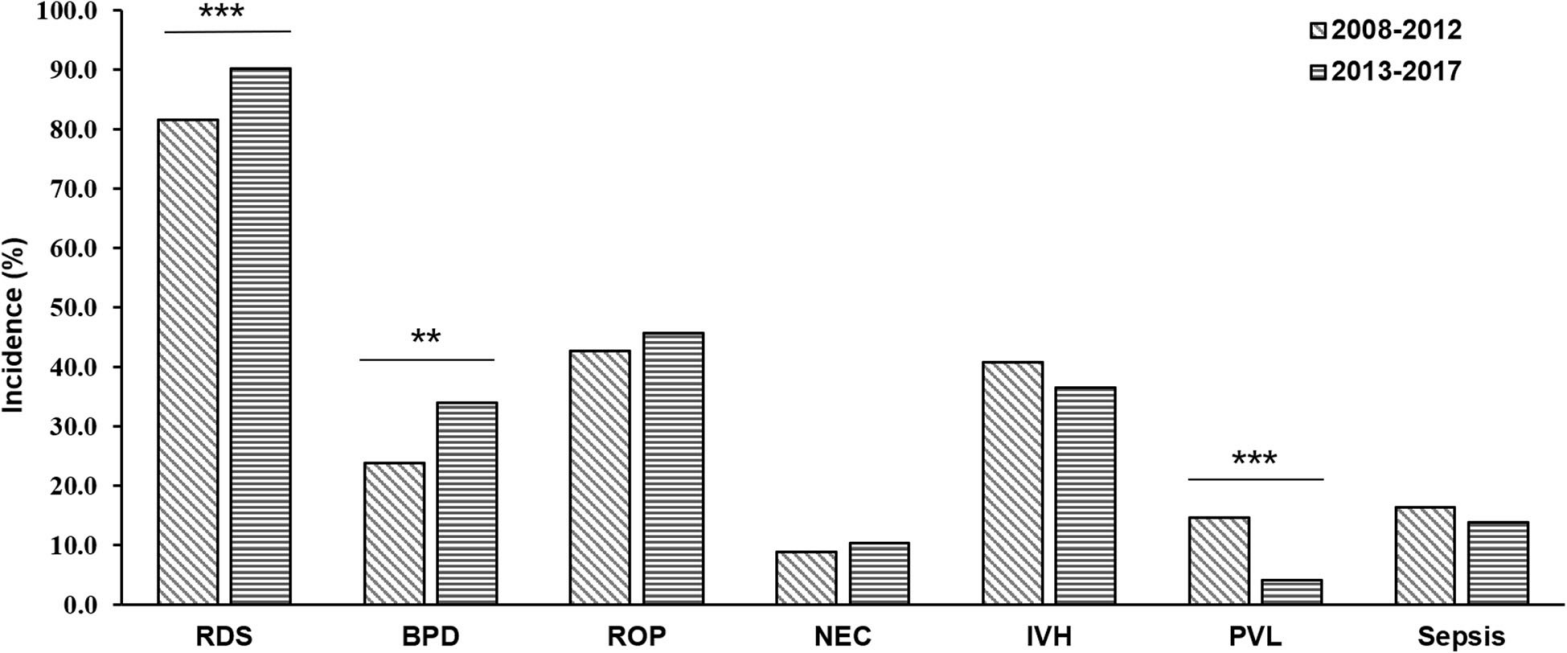
The incidence of complications in EP infants between 2008–2012 and 2013–2017. It was shown that the incidence of RDS and BPD increased during 2013–2017 when compared to 2008–2012 (*p*<0.001 and *p*<0.01, but PVL decreased (*p*<0.001). There were no significant differences in the incidence of ROP, NEC, IVH and Sepsis. RDS: Neonatal respiratory distress syndrome; BPD: Bronchopulmonary dysplasia; ROP: Retinopathy of prematurity; NEC: Necrotizing enterocolitis; IVH: Intraventricular hemorrhage; PVL: Periventricular leukomalacia. Sepsis: blood culture-positive nosocomial sepsis. **:*p*<0.01; ***: *p*<0.001

RDS was the most common complication in EP infants, who were diagnosed with RDS accounted for 88.0% (1804 of 2051). Therefore, the proportion of EP infants receiving surfactant therapy and mechanical ventilation was 78.9% (1618 of 2051) and 78.2% (1603 of 2051) respectively, as shown in Table 1.There was no significant difference in RDS incidence among GA groups as shown in Fig. 1. BPD was another serious complication in EP infants. In this survey, 68.0% (788 of 1158) of EP infants were diagnosed with oxygen dependency on postnatal day 28, but only 32.3% (374 of 1158) still required oxygen therapy at 36 weeks of corrected age or at discharge. The incidence of BPD decreased significantly with the increase of GA (*p* <0.001). 1117 EP infants underwent eye examination, 504 (45.1%) of whom were diagnosed with ROP at different stages, and the proportion of stage ≥3 was 14.6% (163 of 1117). 88 EP infants received further treatments, 40 of whom underwent intravitreal injection with anti-VEGF. The incidence of ROP declined with the increase of GA (*p* <0.001). NEC was evaluated in EP infants who survived for more than 48 h. 10.1% (160 of 1588) of EP infants met the diagnostic criteria at any stage of NEC, 27 of whom required surgery. The incidence of NEC appeared to be positive with GA (*p* <0.05). 1431 EP infants were examined by head ultrasound or MRI, of whom 37.4% (535 of 1431) were diagnosed with IVH, 13.3% (190 of 1431) of EP infants were diagnosed with severe IVH at grade ≥ 3. The incidence of IVH was inversely related to gestational age (*p* <0.001). The incidence of PVL was 6.2% (89 of 1431), and there was no significant difference among GA < 25 weeks, GA = 25 weeks, GA = 26 weeks and GA = 27 weeks. Among the 1588 infants survived more than 48 h, 250 infants (15.7%) were diagnosed with blood culture-positive nosocomial sepsis. The complications for each subgroup of GA are shown in Fig. 1. Additionally, comparing with the period of 2008–2012, the incidence of RDS and BPD increased sharply during the period of 2013–2017(OR = 2.076, 95% CI: 1.574–2.738, *p* < 0.001; OR = 1.645, 95% CI: 1.152–2.351, *p* < 0.01). On the contrary, the incidence of PVL decreased dramatically (OR = 0.252, 95% CI: 0.163–0.392, *p* < 0.001). Moreover, the incidence of ROP, NEC and IVH did not change significantly. The results were shown in Fig. 2.

### Survival days of the non-survivors

**Fig. 3:**
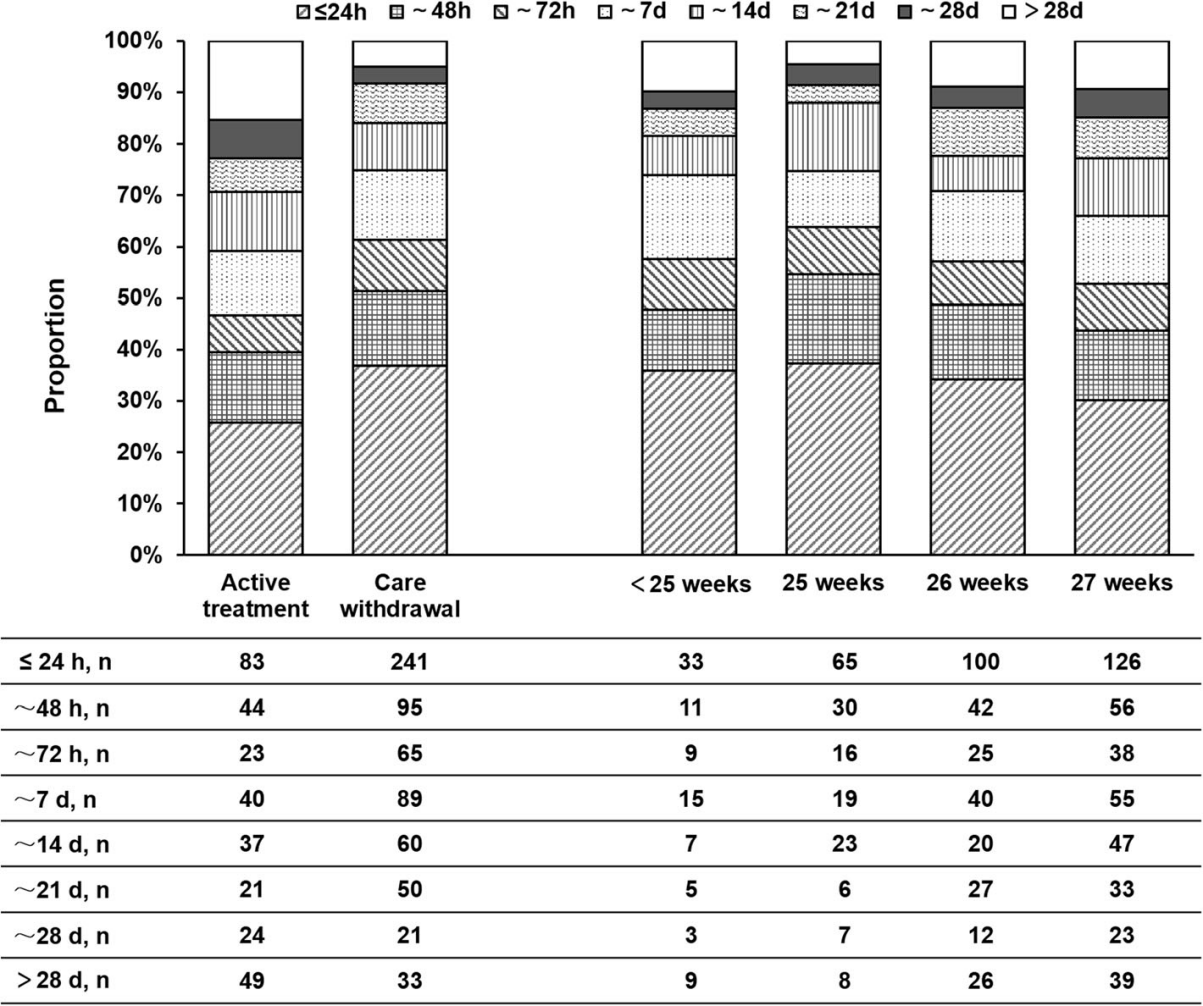
The survival days (hours) of non-survival EP infants. The left part of the chart showed the duration of survival in the non-survival EP infants under active treatment or care withdrawal. The right part of the chart showed the duration of survival in the non-survival EP infants at different GA

Among the 975 non-survivors, 893 (91.6%) of EP infants died during the neonatal period (≤ 28 days), the other 82 (8.4%) died after the neonatal period (> 28 days) as it was shown in Fig. 3. Specifically, 324 (33.2%) of EP infants died on the first day, 139 (14.3%) on the second day, 88 (9.0%) on the third day, 129 (13.2%) during the fourth to seven day, 97 (9.9%) in the second week, 71 (7.3%) in the third week and 45 (4.6%) in the fourth week. The survival days of non-survivors under active treatment or medical care withdrawal were shown in Fig. 3. The chi-square test showed that there was a significant difference in the distribution of survival days between the two groups (*p* < 0.001). No significant difference in the distribution of survival days of non-survivors was found among the different GA (GA < 25 weeks, GA = 25 weeks, GA = 26 weeks or GA = 27 weeks) by chi-square test.

**Fig. 4:**
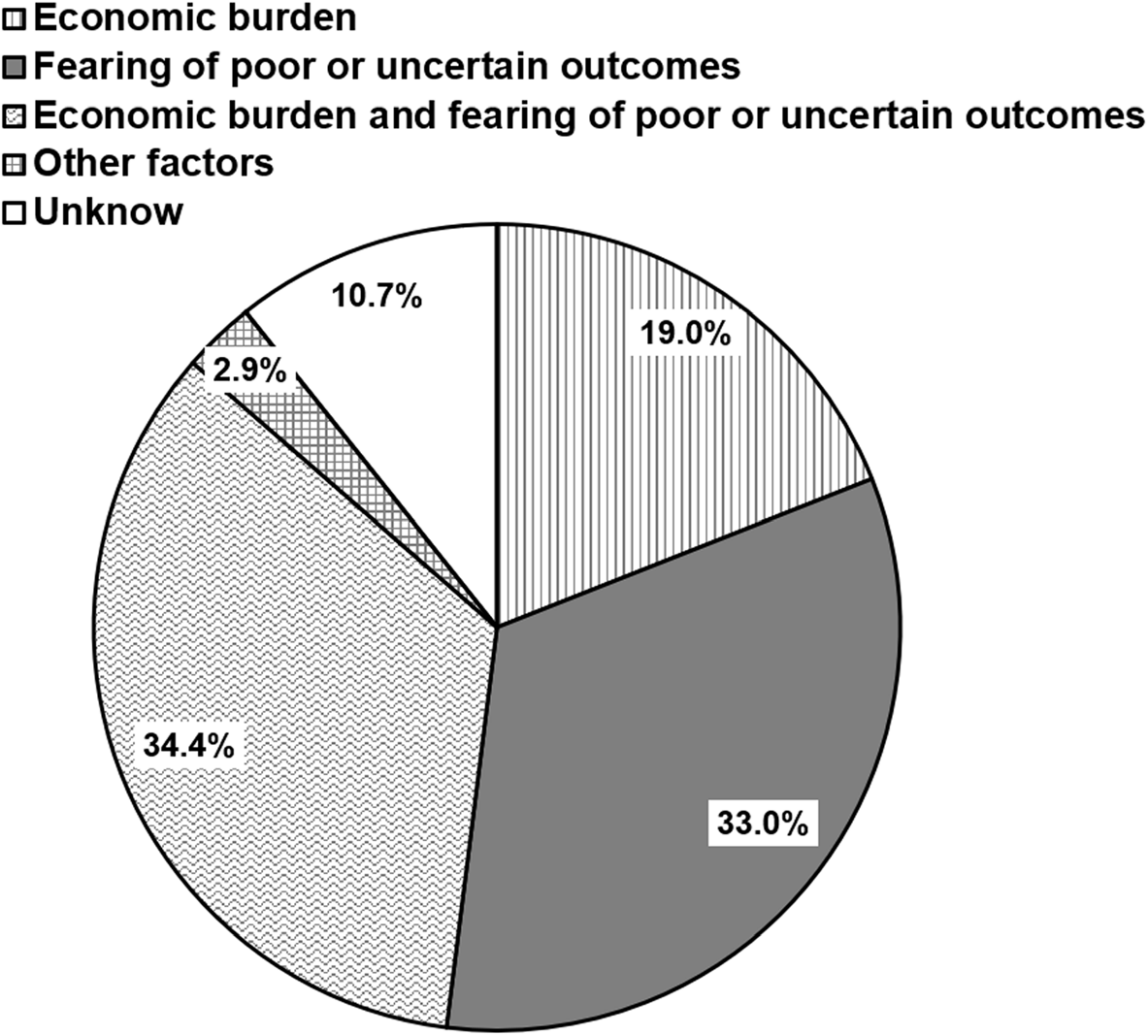
The reasons for medical care withdrawal. Economic burden and fearing of poor or uncertain outcomes, only fearing of poor or uncertain outcomes, only concerning about economic burden, unknow and other factors were the main reasons leading to medical care withdrawal, which was accounted for 34.4, 33.0, 19.0, 10.7 and 2.9% respectively

In our survey, 654 EP infants died mainly as a result of medical care withdrawal. The main reasons for the decision to withdraw care were summarized, as they were shown in Fig. 4. The most important reason was the economic burden and fearing of poor or uncertain outcomes, followed by only fearing of poor or uncertain outcomes, only concerning about economic burden, unknown reason and other factors, which was accounted for 34.4, 33.0, 19.0, 10.7 and 2.7% respectively.

### Multivariate logistic regression analysis of risk factors for survival

As it was shown in Table 5, the multivariate logistic regression analysis indicated that improved survival of EP infants was associated with discharged from specialist hospital, hospitals located in high-level economic development region, increasing gestational age, increasing birth weight, antenatal steroids use and a history of premature rupture of membranes. On the contrary, twins or multiple births, Apgar ≤7 at 5 min, cervical incompetence and decision to withdraw care were associated with decreased survival.

**Table 5:** Multivariate logistic regression analysis of risk factors for survival

Influencing factors	*β* value	Wald *χ*^*2*^	*p*	OR (95% CI)
Specialist hospital	0.820	35.771	<0.001	2.270 (1.735–2.969)
Economic development in high-level	0.301	4.958	0.026	1.351 (1.037–1.762)
Gestational age	0.405	25.770	<0.001	1.499 (1.282–1.753)
Birth weight	0.001	10.020	0.002	1.001 (1.001–1.002)
Twins or multiple births	−0.316	5.288	0.021	0.729 (0.557–0.954)
Apgar ≤7 at 5 min	−0.714	19.088	<0.001	0.490 (0.356–0.675)
Antenatal steroids	0.493	13.999	<0.001	1.637 (1.265–2.119)
Premature rupture of membranes	0.367	5.424	0.020	1.444 (1.060–1.967)
Cervical incompetence	−0.667	4.149	0.042	0.513 (0.270–0.975)
Care withdrawal	−4.490	400.618	<0.001	0.011 (0.007–0.017)

## Discussion

Our study revealed for the first time the short-term outcomes of EP infants in China over 10 years, which provided useful data to gain insight into the current status of preterm infants. A report from WHO [18] declared that preterm birth had become a growing global health issue that the number of preterm infants born worldwide was nearly 15 million annually and continued to grow, and the number of premature babies from China ranked in the second. Despite the encouraging progress, more than a million of preterm infants died mainly because of severe complications associated with premature birth. Prematurity has become the leading cause of death in the first month of life [19]. The Canada Neonatal Network (CNN) data demonstrated substantially decrease in the mortality rate of GA < 29 weeks from 17.2% (1996–1997) to 14.7% (2006–2007) [6]. Isayama T, et al. had compared Neonatal Research Network of Japan (NRNJ) data with CNN data during 2006–2008, the mortality rate at GA < 25 weeks, 26 - 27 weeks, 28–29 weeks and 30–32 weeks were 27.1% vs 52.3%, 9.6% vs 17.9%, 4.1% vs 7.3% and 1.4% vs 1.7% [20]. EP infants with a GA less than 28 weeks are at higher risk of morbidity and mortality. In our survey, the overall mortality of EP infants at discharge was 47.5% during 2008–2017. Specifically, the mortality rate at GA < 24 weeks, 24 weeks, 25 weeks, 26 weeks, and 27 weeks were 88.5, 65.7, 66.2, 53.3, and 37.6% respectively. Although the mortality declined with GA increase, it was obviously higher than in developed countries. It seems that these results may be frustrating, but we were encouraged by the improvement in annual survival rate from 36.2% (21/58) in 2008 to 59.3% (242/408) in 2017.

Due to the definition of preterm birth in China as mentioned above, parents currently can decide whether babies receive treatments or not. Many EP infants died as a result of medical care withdrawal, which accounted for 67.1% in non-survivors of EP infants and 31.9% in total EP infants. What were the factors that influenced the parental decision? Just like in many developing countries, the most important factor may be an economic burden. It was reported recently that cost was indeed an important factor influencing active management of EP infants [21]. As it was indicated in our survey, EP infants in the cities with high level of economic development had low mortality due to medical care withdrawal, such as Guangzhou or Shenzhen. With reference to the annual per capita net income, medical costs for EP infants were a huge burden for families especially in regions of low-level economic development such as cities outside the Pearl Delta. Optimistically, in recent years with an increase in family income and improvement of social security system the survival rate of premature infants has greatly improved.

Moreover, fear of poor or uncertain outcomes in EP infant was another important factor affecting the parental decision. Owing to extreme prematurity, EP infants have a higher incidence of complications, and maybe develop serious consequences in the future [22]. Numerous studies have suggested that major neonatal morbidities are associated with a higher incidence of adverse neuro- developmental outcomes [23–25]. Even worse, due to lack of large samples of epidemiological data for these infants, clinicians or parents were often in the dilemma to make timely and accurate decisions. When expectations were not met or a foreseeable adverse event emerged, parents’ confidence may be shaken and thus the original active treatment options may be changed. In addition, under the influence of outmoded conventional ideas and the only-child policy (the most important part of Family Planning Policy in China), many Chinese families had a preference for male babies, which led to care withdrawal for female infants. In our study, the proportion of male babies was greater than females (61.2%vs 38.9%), but no significant difference was found between the survivor and non-survivor groups.

Extremely preterm birth survivors exhibited significant morbidity. Many studies showed the major neonatal morbidities are predictive of long-term disorders such as motor impairment, cognitive disorders, behavior problems, poor general health, hearing loss, and visual problem. Our study found that the major complications during hospitalization were RDS (88.0%), BPD (32.3%), ROP (45.1%), NEC (10.1%), IVH (37.4%), PVL (6.2%) and blood culture-positive nosocomial sepsis (15.7%). In fact, the incidence of these complications may be much higher than we imagined. There were a considerable number of infants died on account of medical care withdraw whose complications did not develop in our survey. Besides, the diagnosis of some complications may be missed due to lack of relevant equipment for examinations or follow-up data after discharge, especially in the region with low-level economic development. For example, the diagnosis of IVH and PVL might have been missed because they were not checked by head ultrasound or MRI in time. Thus, these results should be interpreted with caution when compared to other studies.

Multiple factors were associated with neonatal mortality and morbidity. A systematic review and meta-analysis showed that antenatal corticosteroid therapy could reduce perinatal death and the incidence of RDS, IVH and NEC in preterm infants [26]. In our study, the survivor group had a higher rate of antenatal corticosteroid therapy than the non-survivor group, but the overall rate of antenatal corticosteroid therapy was lower than other studies [27, 28]. Therefore, prenatal management should be strengthened in the future. In addition, it was indicated in our study that discharged from specialist hospital, discharged hospitals located in high-level economic development region, increasing gestational age, increasing birth weight, antenatal steroids use and a history of premature rupture of membranes were associated with improved survival of EP infants. Instead, twins or multiple births, Apgar ≤7 at 5 min, cervical incompetence and decision to care withdrawal were associated with decreased survival.

Although the total number of subjects in our study was limited, it still represents the short-term outcomes of EP infants in Guangdong province in China. This study was also the first multicenter survey on outcomes of EP infants in China. Therefore, our study may help to delineate the current survival rate of EP infants in China and thus serve as a benchmark for future investigations and studies. Of course, there were still some limitations to our study. First, our survey included only 26 large tertiary hospitals and was neither population- based nor a nationwide study. Second, it only focused on short-term outcomes at discharge and did not consider long term outcomes, especially in neural developmental disabilities.

With societal advancement, economic development and improvements in medicine, as well as adjustments in the Family Planning Policy, the preterm birth has now become an important issue in China. More and more extremely premature infants will be born and survive. To facilitate this, a clear and unified clinical classification and guideline for EP infants should be developed as soon as possible. As in some developed countries, the minimum GA for the definition of preterm birth should be 20–22 weeks. Newborns older than 23 weeks of gestation should routinely receive resuscitation, and even those under 21–22 weeks should be treated similarly if parents insist [29].

## Conclusions

Our study revealed the short-term outcomes of EP infants at discharge among a large population in China for the first time. The survival rate of these infants was lower when compared to developed countries, and medical care withdrawal was a serious dilemma for these infants. Thus, it is crucial to carry on population- based and nationwide research on EP infants in order to develop new clinical practice guidelines for them.

## Data Availability

The datasets used and/or analyzed during the current study are available from the corresponding author on reasonable request.

## Abbreviations

BPD: Bronchopulmonary dysplasia
BW: Birth weight
EP: Extremely preterm
GA: Gestational age
IVH: Intraventricular hemorrhage
MRI: Magnetic resonance imaging
NEC: Necrotizing enterocolitis
NICUs: Neonatal intensive care units
PVL: Periventricular leukomalacia
RDS: Respiratory distress syndrome
SD: Standard deviation
